# Th2 and eosinophil responses suppress inflammatory arthritis

**DOI:** 10.1038/ncomms11596

**Published:** 2016-06-07

**Authors:** Zhu Chen, Darja Andreev, Katharina Oeser, Branislav Krljanac, Axel Hueber, Arnd Kleyer, David Voehringer, Georg Schett, Aline Bozec

**Affiliations:** 1 Department of Internal Medicine 3, University Hospital Erlangen and Friedrich Alexander University of Erlangen-Nuremberg, Gluckstrasse 6, Erlangen 91054, Germany; 2 Department of Rheumatology and Immunology, Anhui Medical University Affiliated Provincial Hospital, Hefei 230001, China; 3 Department of Infection Biology, University Hospital Erlangen and Friedrich-Alexander University Erlangen-Nuremberg (FAU), Erlangen 91054, Germany

## Abstract

Th2–eosinophil immune responses are well known for mediating host defence against helminths. Herein we describe a function of Th2–eosinophil responses in counteracting the development of arthritis. In two independent models of arthritis, *Nippostrongylus brasiliensis* infection leads to Th2 and eosinophil accumulation in the joints associated with robust inhibition of arthritis and protection from bone loss. Mechanistically, this protective effect is dependent on IL-4/IL-13-induced STAT6 pathway. Furthermore, we show that eosinophils play a central role in the modulation of arthritis probably through the increase of anti-inflammatory macrophages into arthritic joints. The presence of these pathways in human disease is confirmed by detection of GATA3-positive cells and eosinophils in the joints of rheumatoid arthritis patients. Taken together, these results demonstrate that eosinophils and helminth-induced activation of the Th2 pathway axis effectively mitigate the course of inflammatory arthritis.

## Abstract (web summary)

Type 2 immune responses are viewed as opposites of Type 1 and 17 responses. Here the authors show that activation of Type 2 immunity by helminth infection counteracts the development of inflammatory arthritis, a type 17-mediated pathology, via IL-4/IL-13- STAT6 signalling and eosinophil activation.

## Graphical abstract

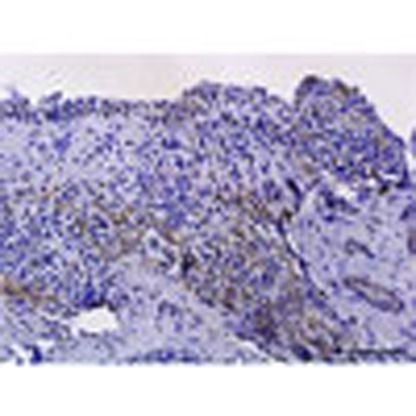

Rheumatoid arthritis (RA) is a chronic autoimmune disease characterized by synovial inflammation and bone erosion, which affects up to 1% of the population worldwide. It is the paradigm of a chronic disease, which hardly resolves and usually accompanies patients during their entire life^1^,^2^. This situation is particularly challenging as RA can start early in life, even affecting children. The induction of type 1 (Th1) and type 17 (Th17) helper T-cell responses in the context of underlying autoimmunity are considered to play a key role in the initiation phase of RA^1^. Once inflammation is established in arthritis, the process turns out to be highly chronic without evidence for spontaneous resolution. Although the pro-inflammatory pathways in RA are well understood, immune mechanisms counteracting inflammation are yet to be characterized. Hence, finding intrinsic regulatory pathways fostering the impaired resolution processes in arthritis is of utmost importance. Discovery of such pathways may not only be critical in elucidating the pathophysiology of RA but may also uncover some general principles why resolution is impaired in chronic inflammatory diseases.

Type 2 (Th2) immune responses are induced by helminth infection in mice and in humans, and participate in the resolution of inflammation and wound healing^3^,^4^,^5^. Helminth infections are found in ∼1 billion people worldwide, mainly in underdeveloped countries. Severe infections are rare and adults suffer of mild or asymptomatic disease. Helminths such as *N. brasiliensis* trigger robust Th2 responses *in vivo*, characterized by increased production of Th2 cytokines such as interleukin (IL)-4 and IL-13, accompanied with activation and expansion of CD4^+^Th2 cells, eosinophilia, goblet and mucosal mast cell hyperplasia^6^,^7^,^8^,^9^,^10^,^11^. On the other hand, helminth infections also foster anti-inflammatory signals by inducing production of IL-10, Foxp3^+^ regulatory T cells and alternatively activated macrophages^12^,^13^,^14^,^15^,^16^, which suppresses immune activation in the gut, the lungs and in autoimmune diabetes^14^,^15^,^17^. Hence, helminth infection and associated Th2 responses may represent a promising pathway for fostering the resolution of arthritis. The exact mechanism, how such induction of Th2 responses by helminths infection could modulate arthritis, however, is still unknown.

Herein, we show that *N. brasiliensis* infection alleviated disease in two models of inflammatory arthritis. At the molecular level, the attenuation of arthritis is dependent on IL-4/IL-13 secretion and STAT6 signalling pathway in haematopoietic cells. Moreover, hypereosinophilia triggered by *N. brasiliensis* infection also contributes to the resolution of arthritis by stimulating a shift from pro- into anti-inflammatory macrophages in the joint. Hence, our findings indicate a crucial role of Th2 immune responses in inhibiting the development of arthritis.

## Results

### *N. brasiliensis* infection suppresses inflammatory arthritis

**Figure 1:**
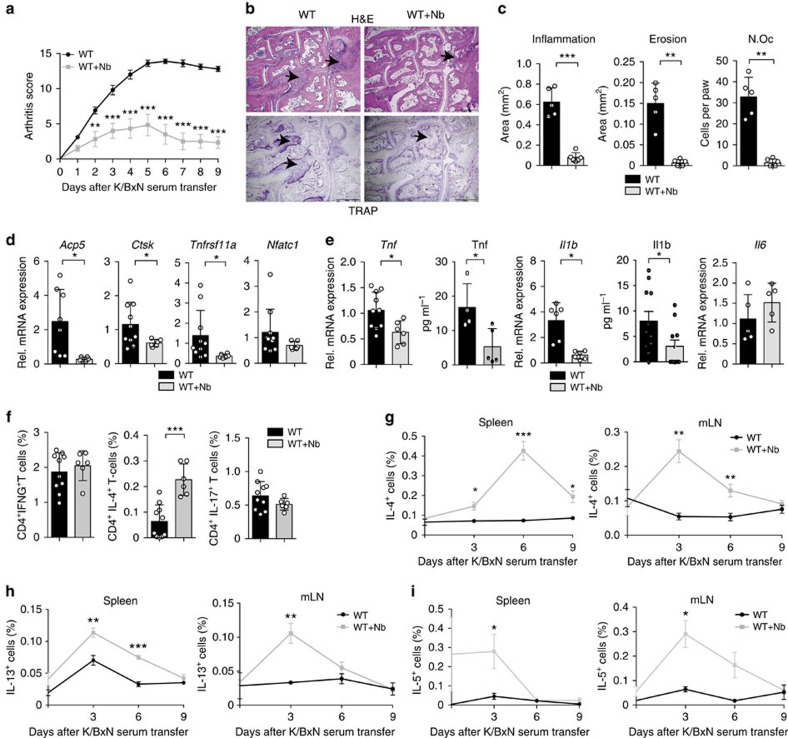
*N. brasiliensis* infection inhibits K/BxN SIA. (**a**) Arthritis scores from WT mice infected or not with *N. brasiliensis* (Nb) (*n*=6–10 per group). (**b**,**c**) Hematoxylin and eosin (H&E) and tartrate-resistant acid phosphatase (TRAP) staining (**b**), and quantification of inflammation area, erosion area and number of osteoclasts per paw (N.Oc per paw) (**c**) in the hind paw of WT mice with or without Nb challenge at day 9 post serum transfer (*n*=6 per group). Scale bar, 500 μm. (**d**,**e**) Analyses of *Acp5* (encoding TRAP), *Ctsk* (encoding cathepsin K), *Tnfrsf11a* (encoding RANK), *Nfatc1* (encoding NFATc1) (**d**), *Tnf*, *Il1b* and *Il6* (**e**) mRNA expression in synovial extracts and TNF and IL1β serum levels in WT mice with and without Nb challenge at day 9 post serum transfer (*n*=8–11 per group). (**f**) Frequency of CD4^+^IFN-γ^+^ (Th1), CD4^+^IL-4^+^ (Th2) and CD4^+^IL-17^+^ (Th17) cells in the spleen of WT mice with or without Nb challenge at day 9 post serum transfer (*n*=6–10 per group). (**g**–**i**) Frequency of IL-4^+^ (**g**), IL-13^+^ (**h**) and IL-5^+^ (**i**) lymphocytes in the spleen and in mesenteric lymph node (mLN) from WT mice with or without Nb challenge at the indicated time points (*n*=3–5 per group). Data are shown as mean±s.e.m. Pictures are representative of 3 independent experiments. Asterisks mark statistically significant difference (*P<0.05, **P<0.01 and ***P<0.001 determined by Student's *t*-test).

To assess the role of Th2 responses in arthritis, we performed serum-induced arthritis (SIA) in mice that were either untreated or infected with *N. brasiliensis*. Interestingly, infected mice displayed a significant reduction of arthritis as compared with non-infected controls (Fig. 1a). Histologic analyses of the hind paws showed decreased inflammation scores and protection from bone destruction in *N. brasiliensis*-infected compared with non-infected mice (Fig. 1b,c). In accordance, expression levels of osteoclast markers and pro-inflammatory cytokines were decreased in the joints of *N. brasiliensis*-infected compared with control mice (Fig. 1d,e), suggesting an effective inhibition of arthritis by worm infection.

### *N. brasiliensis* inhibited arthritis associated with Th2 cells

To confirm that *N. brasiliensis* infection conducts a Th2-biased immune response, T-cell subsets were quantified in the spleen of *N. brasiliensis*-infected mice and non-infected controls. Although CD4^+^ IFN-γ^+^ (Th1) and CD4^+^IL-17^+^ (Th17) cells remained unchanged after *N. brasiliensis* infection, CD4^+^IL-4^+^ (Th2) cells significantly increased in *N. brasiliensis*-infected compared with non-infected mice (Fig. 1f). To characterize the kinetics of the Th2 response, cellular IL-4, IL-5 and IL-13 expression levels were assessed at day 0, 3, 6 and 9 after induction of arthritis. IL-4- and IL-13-positive cells increased up to day 9 post *N. brasiliensis* infection and remained high in the spleen and lymph nodes of *N. brasiliensis*-infected mice (Fig. 1g,h). Furthermore, IL-5-producing cells were increased already early and returned to baseline level 6 days after induction of arthritis (Fig. 1i). All together, these data showed a profound Th2 response after *N. brasiliensis* challenge during the onset of arthritis.

### *N. brasiliensis* triggers eosinophil accumulation in arthritic joint

**Figure 2:**
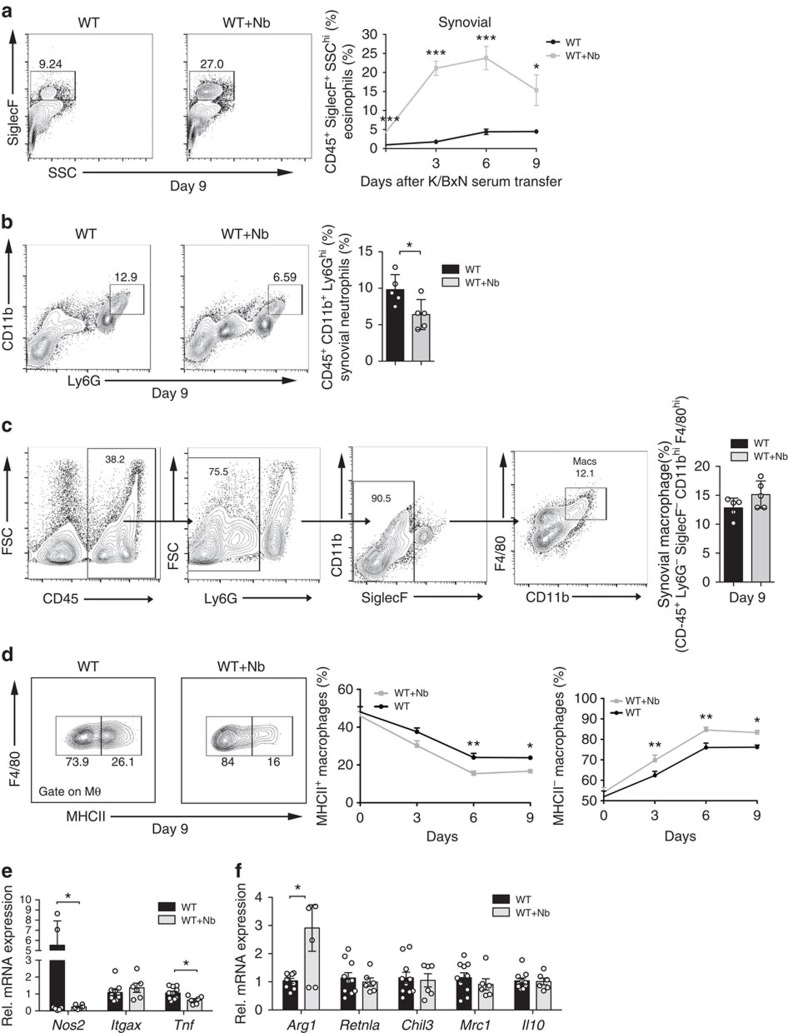
*N. brasiliensis* infection increases eosinophil and anti-inflammatory macrophage numbers in the joints. (**a**) Representative contour plots and eosinophil (CD45^+^SiglecF^+^) quantification in the ankle joint of WT mice with or without *N. brasiliensis* (Nb) challenge at day 9 post serum transfer (*n*=5 per group). Numbers represent percentage in CD45^+^ cells. Frequency of eosinophils in the joints of WT mice with or without Nb challenge at the indicated time points (*n*=5 per group). (**b**) Representative contour plots and percentage of neutrophil (CD45^+^CD11b^+^Ly6G^hi^) in the joints of WT mice with or without Nb challenge at day 9 post serum transfer (*n*=5 per group). (**c**) Gating strategy for the identification of macrophages in the joints of WT mice. Percentage of total macrophages (defined as CD45^+^Ly6G^−^SiglecF^−^CD11b^hi^F4/80^hi^ cells) in WT mice with or without Nb challenge 9 day post serum transfer (*n*=5 per group). Numbers represent percentage of the parent population. (**d**) Frequency of pro (MHC II^+^) and anti-inflammatory (MHC II^−^) macrophages in the joints of WT mice with or without Nb challenge at the indicated time points (*n*=15 per group). (**e**,**f**) Quantitative reverse transcriptase–PCR (RT–PCR) analyses of (**e**) *Nos2* (encoding inducible nitric oxide synthase), *Itgax* (encoding CD11c) and *Tnf*, as well as (**f**) *Arg1* (encoding arginase-1), *Retnla* (encoding resistin-like alpha), *Chil3* (encoding chitinase-like protein 3), *Mrc1* (encoding macrophage mannose receptor 1) and *Il10* expression in synovial extracts from WT mice with or without Nb challenge (*n*=6–10 per group). Data are shown as mean±s.e.m. Pictures are representative of 3 independent experiments, except for (**d**) where the data were pooled from three independent repeats. Asterisks mark statistically significant difference (*P<0.05, **P<0.01 and ***P<0.001, determined by Student's *t*-test).

To examine whether *N. brasiliensis*-triggered Th2 responses induce respective effector cell accumulation, eosinophils, neutrophils and macrophages were quantified in the joints of non-infected and *N. brasiliensis*-infected mice. We set up the fluorescence-activated cell sorting strategy for quantifying eosinophils as CD45^+^Ly6G^−^CD11b^+^SiglecF^+^-positive cells using ΔdblGATA mice for the negative control setting (Supplementary Fig. 1a). We could demonstrate that eosinophil numbers increased in the joint of *N. brasiliensis*-infected mice. Time-course analyses revealed a rapid increase of eosinophils during the onset of arthritis (Fig. 2a). In contrast, neutrophils were decreased in the joints of *N. brasiliensis*-infected mice (Fig. 2b). To define whether Th2–eosinophil induction also affects macrophage patterning in the joints, the percentages of total and anti-inflammatory macrophages were quantified in the joints. Although macrophages defined as CD45^+^Ly6G^−^SiglecF^−^CD11b^hi^F4/80^hi^ cells were unaffected in the joints of *N. brasiliensis*-infected mice (Fig. 2c), the proportion of previously described^18^ anti-inflammatory major histocompatibility complex (MHC) II^−^ macrophages increased, while pro-inflammatory MHC II^+^ macrophages decreased in the joints of *N. brasiliensis*-infected arthritic mice (Fig. 2d). Time-course analyses revealed a rapid increase of anti-inflammatory MHC II^−^ macrophages in the joints of *N. brasiliensis*-infected mice (Fig. 2d). In accordance with the switch of the macrophage status, messenger RNA profiling of pro- and anti-inflammatory markers showed decreased *Nos2* and *Tnf* but increased *Arg1* levels (Fig. 2e,f). These data showed that *N. brasiliensis*-induced Th2 responses affect the cellular composition of the arthritic joint with increased numbers of eosinophils and anti-inflammatory macrophages.

### Consistent effects of *N. brasiliensis* in *hTNFtg* mice

**Figure 3:**
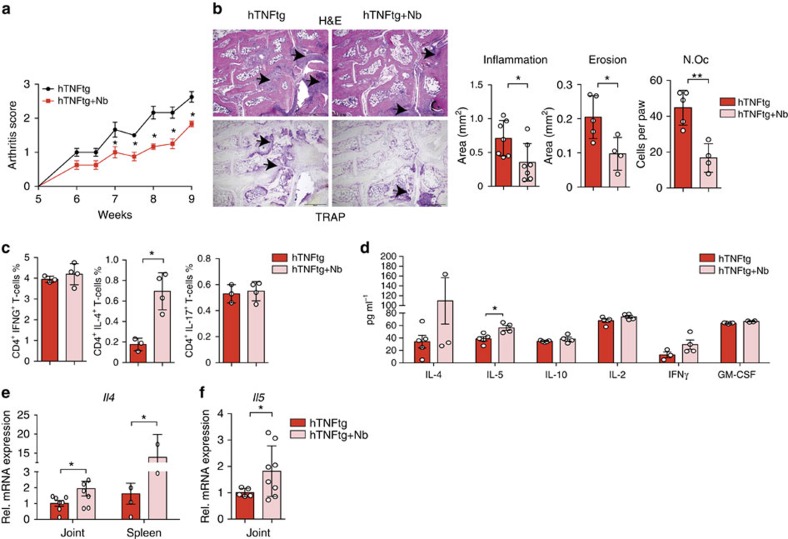
*N. brasiliensis* infection induces Th2 response and alleviates arthritis in TNFα- mediated arthritis. (**a**) Arthritis score from 5-week-old hTNFtg mice with or without *N. brasiliensis* (Nb) challenge. Data are pooled from two independent experiments (*n*=9 per group). (**b**) Haematoxylin/eosin (H&E) and tartrate-resistant acid phosphatase (TRAP) staining of the hind paw from 9-week-old hTNFtg mice with or without Nb challenge. Scale bar, 500 μm. Quantification of inflammation area, erosion area and osteoclast number (N.Oc) per paw in the hind paws of hTNFtg mice with or without Nb challenge (*n*=5–8 per group). (**c**) Frequency of CD4^+^IFN-γ^+^ (Th1), CD4^+^IL-4^+^ (Th2) and CD4^+^IL-17^+^ (Th17) cells in the spleen of hTNFtg mice with or without Nb challenge (*n*=4–5 per group). (**d**) Serum levels of IL-4, IL-5, IL-10, IL-2, IFN-γ and granulocyte–macrophage colony-stimulating factor (GM-CSF) in hTNFtg mice with or without Nb challenge (*n*=4–5 per group). (**e**) Quantitative reverse transcriptase–PCR (RT–PCR) analyses of *Il4* expression in the spleen and synovial extracts from 9-week-old hTNFtg mice with or without Nb challenge (*n*=4–8 per group). (**f**) Quantitative RT–PCR analyses of *Il5* expression in synovial extracts of hTNFtg mice with or without Nb challenge (*n*=4–8 per group). Data are expressed as mean±s.e.m. Pictures are representative of 3 independent experiments. Asterisks mark statistically significant difference (*P<0.05 and **P<0.01 determined by Student's *t*-test).

To confirm the inhibitory effect of *N. brasiliensis*-induced Th2 responses on arthritis, we examined a second model of arthritis and challenged 5-week-old human tumour necrosis factor (TNF) transgenic (*hTNFtg*) mice with *N. brasiliensis*. Similar to the findings in SIA model, *N. brasiliensis*-infected *hTNFtg* mice displayed mitigated arthritis compared with non-infected controls (Fig. 3a). Histopathological analyses revealed decreased articular inflammation, less bone erosion and a lower number of osteoclasts in the paws of *N. brasiliensis*-challenged mice compared with controls (Fig. 3b). Furthermore, osteoclast numbers and sizes were also lower in the tibia of *N. brasiliensis*-challenged *hTNFtg* mice (Supplementary Fig. 2a). Expression of osteoclast markers such as *Acp5*, but not *Tnfrsf11a*, *Ctsk* or *Nfatc1* were found decreased (Supplementary Fig. 2b). Micro-computed tomography analysis of the tibial bones revealed an increased bone volume and trabecular number in *N. brasiliensis*-infected *hTNFtg* mice (Supplementary Fig. 2c,d). Taken together, these data showed that infection with *N. brasiliensis* also ameliorated TNFα-mediated joint inflammation and the resulting bone loss. Analysis of the immune response showed significantly increased frequency of CD4^+^IL-4^+^ (Th2) cells without affecting CD4^+^ IFN-γ^+^ (Th1) and CD4^+^IL-17^+^ (Th17) cells (Fig. 3c) in *hTNFtg* mice challenged with *N. brasiliensis*. Accordingly, IL-4 and IL-5 protein levels were increased in the serum (Fig. 3d). In addition, mRNA expression of *Il4* and *Il5* in the joints and the spleen of *N. brasiliensis*-infected hTNFtg mice was increased compared with non-infected mice (Fig. 3e,f), confirming the induction of Th2 responses in the joints after *N. brasiliensis* infection.

### *N. brasiliensis* attenuated arthritis is IL-4/IL-13 dependent

**Figure 4:**
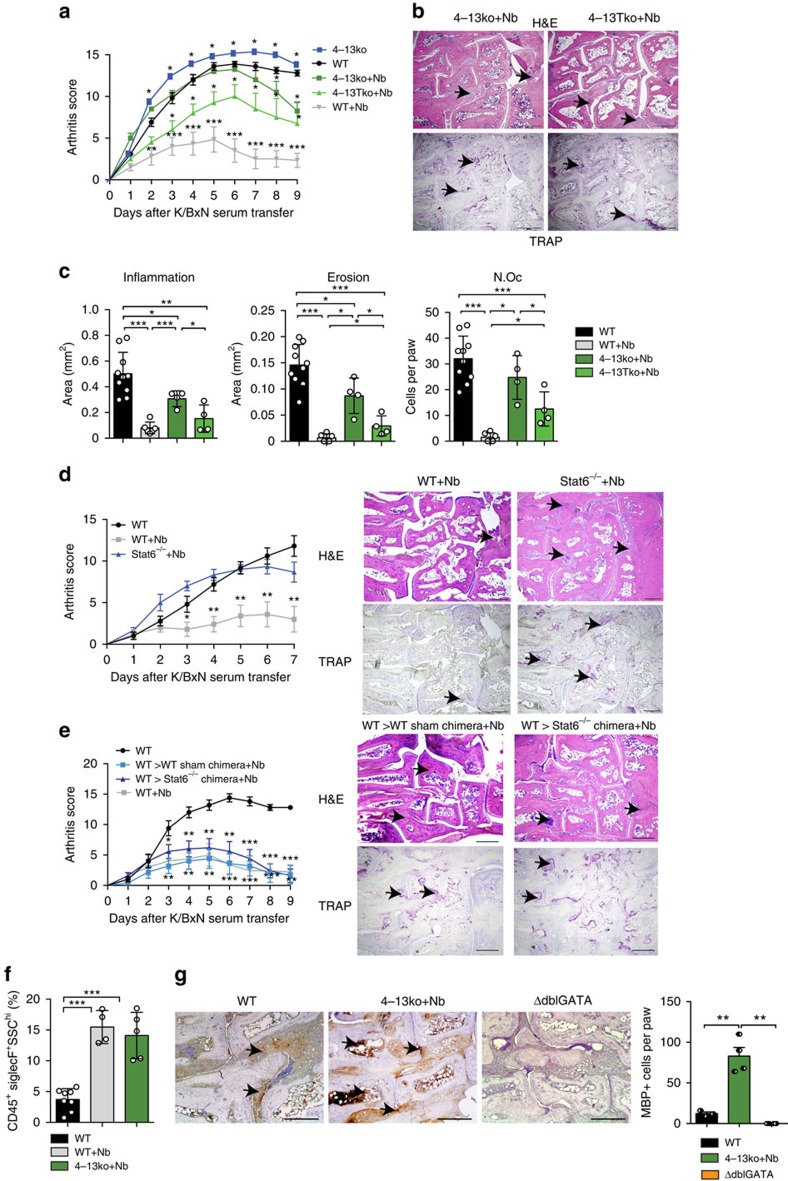
Attenuation of arthritis by *N. brasiliensis* depends on the activation of the IL-4/IL-13/STAT6 pathway. (**a**) Arthritis score from WT, 4–13ko and 4–13Tko with or without *N. brasiliensis* (Nb) challenge (*n*=4–6 per group). (**b**,**c**) Haematoxylin/eosin (H&E) and tartrate-resistant acid phosphatase (TRAP) staining and quantification of inflammation area, erosion area and number of osteoclasts (N.Oc) per paw on day 9 post K/BxN serum transfer in the indicated group of mice (*n*=4–6 per group); scale bar, 500 μm. (**d**) Arthritis scores and corresponding H&E and TRAP stainings in unchallenged WT mice and Nb-challenged WT and *Stat6*^−/−^ mice (*n*=3–5 per group); scale bar, 500 μm. (**e**) Arthritis score in unchallenged WT mice and Nb-challenged WT=>WT and WT=>*Stat6*^−/−^ chimeras (*n*=5 per group); scale bar, 500 μm. (**f**) Percentage of eosinophils on day 9 post serum transfer in unchallenged WT and Nb-challenged WT and 4–13ko mice (*n*=4–8 per group). (**g**) Major Basic protein (MBP) staining in the joints of WT, Nb-challenged 4–13ko mice and eosinophil-deficient ΔdblGATA mice (negative control) on day 9 post K/BxN serum transfer and respective quantification of staining. All data are expressed as mean±s.e.m. Pictures are representative of 3 independent experiments. Asterisks mark statistically significant difference (*P<0.05, **P<0.01 and ***P<0.001 determined by Student's *t*-test for single comparison (**a**,**d**,**e** compared with WT without infection) or analysis of variance test for multiple comparisons (**c**,**f**,**g**)).

To explore the molecular mechanisms involved in the anti-arthritic effect of *N. brasiliensis* infection, we challenged *Il4*^−/−^*Il13*^−/−^ (4–13ko) mice and T cell-specific *Il4*^−/−^*Il13*^−/−^ (4–13Tko) mice with *N. brasiliensis* before the induction of arthritis. Without *N. brasiliensis* infection, the level of arthritis was increased in *4–13ko* mice compared with wild-type (WT) controls (Fig. 4a). Moreover, the beneficial effect of *N. brasiliensis* infection on the onset of arthritis was virtually abolished in *4–13ko* and *4–13Tko* mice, showing almost identical induction of arthritis similar to that in the non-infected controls (Fig. 4a). However, in contrast to WT controls some resolution of arthritis was still observed in *Nb*-challenged *4–13ko* and *4–13Tko* mice, suggesting additional regulatory processes being involved. This concept was also confirmed by the histopathological analysis of arthritis scores, bone erosion scores and osteoclast numbers (Fig. 4b,c). Effects on bone and osteoclasts reflected the effects on inflammation, whereas no intrinsic differences in osteoclastogenesis and osteoclast gene expression were observed between WT and *4–13ko* monocyte lineage cells (Supplementary Fig. 3a,b).

### *N. brasiliensis*-attenuated arthritis is STAT6 dependent

These data suggested that IL-4/IL-13 pathway is important for mediating the inhibitory effects of *N. brasiliensis* infection on arthritis. As STAT6 signalling pathway is essential for mediating IL-4/IL-13-induced response^19^, we also induced arthritis in *N. brasiliensis*-infected *Stat6*^−/−^ mice. *N. brasiliensis*-infected *Stat6*^−/−^ mice developed comparable SIA than non-infected mice (Fig. 4d and Supplementary Fig. 4a), confirming the dependence of the STAT6 signalling pathway in the mitigation of arthritis. To further evaluate the role of the STAT6-regulated genes in haematopoietic cells, *Stat6*^−/−^ and WT recipient mice were reconstituted with WT bone marrow. These chimeric mice were then challenged with *N. brasiliensis* and induced for arthritis. Indeed, WT=>*Stat6*^−/−^ and WT=>WT chimeras displayed similar inhibition of arthritis by *N. brasiliensis* (Fig. 4e and Supplementary Fig. 4b), suggesting that inhibitory effects depend on STAT6-regulated genes in haematopoietic cells.

### Hypereosinophilia reduced arthritis in 4–13ko-infected mice

Although the induction phase of arthritis was comparable, disease partly resolved in the later stages of *N. brasiliensis* infected *4–13ko* and *Stat6*^−/−^ mice (Fig. 4a–d). As the *N. brasiliensis*-induced increase of anti-inflammatory macrophages in the joints was abolished in *4–13ko* mice (Supplementary Fig. 4c), other immune cells seem to be responsible, for this enhances resolution of arthritis in *N. brasiliensis*-infected *4–13ko* mice. As induction of eosinophils is a hallmark of helminth infection, peripheral eosinophil frequency was measured during this resolution phase, 9 days after induction of arthritis. Significant higher eosinophils were found in both *N. brasiliensis*-infected WT, but also *4–13ko* mice compared with non-infected controls (Fig. 4f). In addition, increased eosinophil infiltration of the joints of *4–13ko* mice was noticed when staining for the eosinophil marker major basic protein (Fig. 4g). Taken together, these findings suggest that eosinophils have an intrinsic inhibitory effect on the disease course of arthritis.

### Eosinophil deficiency exacerbates arthritis

**Figure 5:**
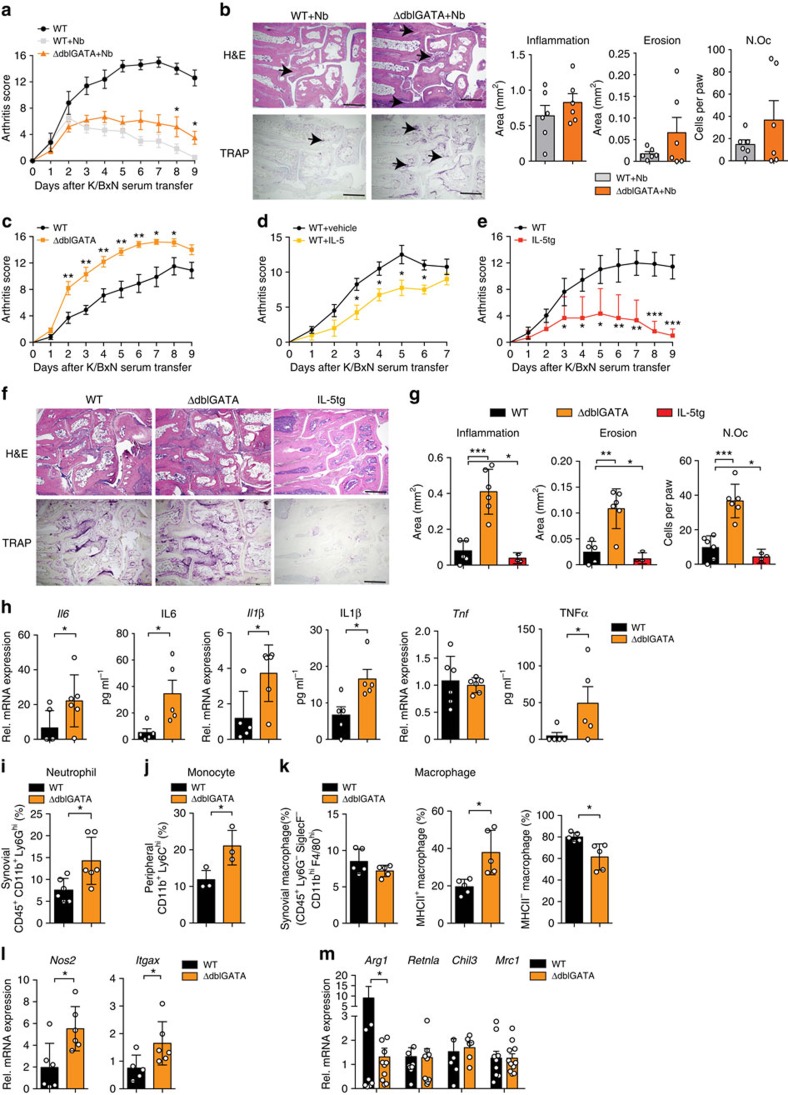
Eosinophil numbers control resolution of arthritis. (**a**) Arthritis score in WT unchallenged and *N. brasiliensis* (Nb) challenged WT mice and eosinophil-deficient ΔdblGATA mice induced for K/BxN serum transfer arthritis (*n*=6 per group). (**b**) Haematoxylin/eosin (H&E) and tartrate-resistant acid phosphatase (TRAP) staining and quantification of inflammation area, erosion area and number of osteoclasts (Oc.N) per paw 9 days after serum transfer (*n*=6 per group); scale bar, 500 μm. (**c**) Arthritis score in WT and ΔdblGATA mice after K/BxN serum transfer (*n*=6 per group). (**d**) Arthritis scores in WT mice injected with vehicle or recombinant IL-5 during serum transfer (*n*=4 per group). (**e**) Arthritis scores in WT and IL-5tg mice after serum transfer (*n*=6 per group). (**f**) H&E and TRAP staining and (**g**) quantification of inflammation area, erosion area and N.Oc per paw 9 days after serum transfer (*n*=6 per group); scale bar, 500 μm. (**h**) Analyses of *Il6, Il1β* and *Tnfα* mRNA expression in synovial extracts and IL6, IL1β and TNFα serum level of WT and ΔdblGATA mice 9 days after serum transfer (*n*=6 per group). (**i**) Percentage of CD45^+^CD11b^+^Ly6G^hi^ neutrophils in the joints of arthritic WT and ΔdblGATA mice (*n*=5 per group). (**j**) Percentage of peripheral CD11b^+^Ly6C^hi^ monocytes in arthritic WT and ΔdblGATA mice (*n*=3 per group). All analyses above were performed 9 days after serum transfer. (**k**) Percentage of total macrophages (CD45^+^Ly6G^−^SiglecF^−^CD11b^hi^F4/80^hi^ cells), MHC II^+^ macrophages and MHC II^−^ macrophages in the joints of WT and ΔdblGATA mice 9 day after serum transfer (*n*=5 per group). (**l**) Quantitative reverse transcriptase–PCR (RT–PCR) analyses of *Nos2* and *Itgax* expression in joint extracts of WT and ΔdblGATA arthritic mice (*n*=6 per group). (**m**) Quantitative RT–PCR analyses of *Arg1*, *Retnla*, *Chil3* and *Mrc1* expression in joint extracts of WT and ΔdblGATA mice 9 days after serum transfer (*n*=6–11 per group). Data are expressed as mean±s.e.m. Pictures are representative of 3 independent experiments. Asterisks mark statistically significant difference (*P<0.05, **P<0.01 and ***P<0.001 determined by Student's *t*-test for single comparison).

To further address the role of eosinophils in arthritis, controls, eosinophil-deficient ΔdblGATA mice and eosinophil-overexpressing *Il5*tg mice were induced for SIA. In addition, recombinant IL-5, known to induce eosinophils, was injected into WT mice before and during induction of arthritis. Strikingly, arthritis in *N. brasiliensis*-challenged eosinophil-deficient ΔdblGATA mice was milder than in non-infected controls but lacked the resolution phase observed in WT mice challenged with *N. brasiliensis* (Fig. 5a,b). The absence of eosinophils was associated with a shift from anti- to pro-inflammatory macrophages in the joints (Supplementary Fig. 5a,b). Furthermore, ΔdblGATA mice showed significantly enhanced severity of arthritis *per se* (Fig. 5c). Re-introduction of eosinophils into ΔdblGATA mice during the course of arthritis stimulated initiation of resolution and reduced arthritis scores to the levels observed in WT mice (Supplementary Fig. 5c,d). In contrast, *Il5*tg mice and, to a lesser extent, also IL-5 treatment of WT mice led to significant reduction of arthritis scores (Fig. 5d,e). Accordingly, the extent of inflammation, bone erosion and number of osteoclasts were increased in ΔdblGATA mice and decreased in *Il5*tg mice (Fig. 5f,g). Furthermore, pro-inflammatory cytokines such as *Il6* and *Il1β* were increased at mRNA and protein levels in ΔdblGATA arthritic mice compared with WT controls (Fig. 5h), suggesting that eosinophils play an important role in determining the severity of arthritis.

Next, the cellular environment in the joints of eosinophil-deficient arthritic mice was assessed: neutrophil numbers and neutrophil-associated chemokines *Cxcl1* and *Cxcl2* were significantly higher in the joints of arthritic *ΔdblGATA* mice compared with WT controls (Fig. 5i and Supplementary Fig. 5e). Peripheral Ly6C^hi^ inflammatory monocytes were also higher in *ΔdblGATA* mice than in controls (Fig. 5j), correlating with increased *Ccr2* expression in the synovium of *ΔdblGATA* mice (Supplementary Fig. 5f). The increased peripheral Ly6C^hi^ inflammatory monocytes might promote the expression of neutrophil-associated chemokines. Moreover, in the joints pro-inflammatory MHC II^+^ macrophages increased, while anti-inflammatory MHC II^−^ macrophages decreased in *ΔdblGATA* mice compared with WT controls (Fig. 5k), correlating with decreased expression of *Arg1* and increased expression of *Nos2* and *Itgax* (Fig. 5l,m). These data show that the absence of eosinophils changes the balance between pro- and anti-inflammatory macrophages increasing neutrophils recruitment and leading to exacerbated inflammation.

### Th2 cells and eosinophils in human arthritic joints

**Figure 6:**
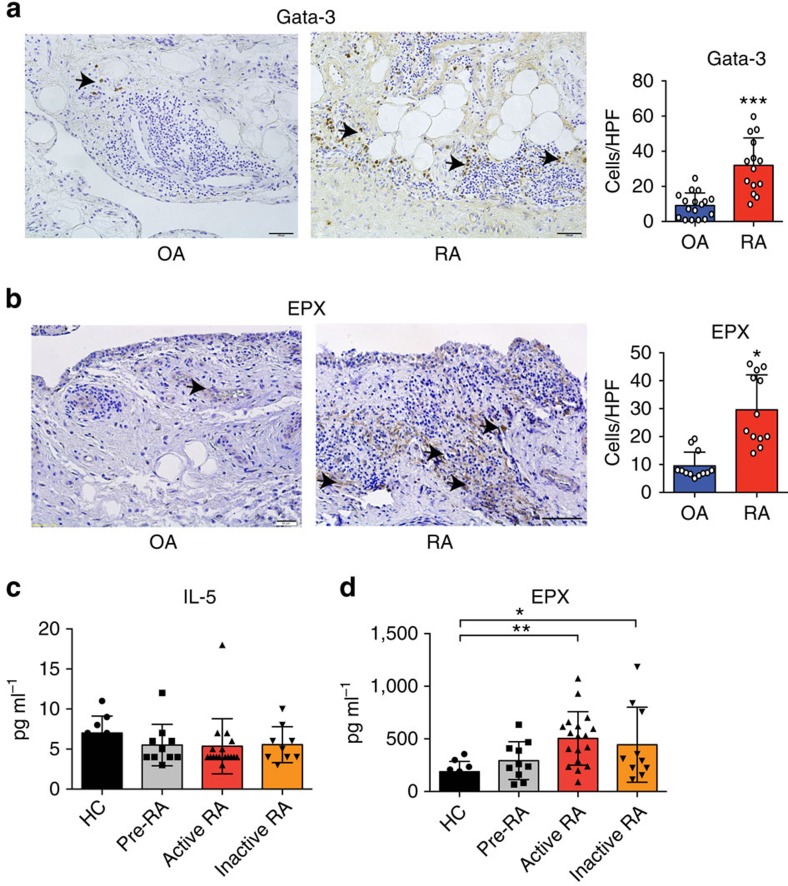
Expression of Th2 and eosinophil markers in human rheumatoid arthritis. (**a**) Representative immunohistochemistry staining of GATA3 in the synovium of osteoarthritis (OA) and RA patients. Positive cells per high-power field were compared between groups (*n*=17 OA patients and 14 RA patients). (**b**) Representative immunohistochemistry staining of EPX in the synovium of OA and RA patients. Positive cells per high-power field were compared between groups (*n*=12 OA patients and 12 RA patients). (**c**) IL-5 serum levels in healthy controls, autoantibody-positive individuals without RA, active and inactive RA patients (*n*>10 patients per group). (**d**) Serum EPX levels in healthy controls, autoantibody-positive individuals without RA, active and inactive RA patients (*n*>10 patients per group). (*P<0.05, **P<0.01 and ***P<0.001 determined by Student's *t*-test for single comparison (**a**,**b**) or analysis of variance test for multiple comparisons (**c**,**d**)).

To define whether the Th2 response could also occur in human inflammatory arthritis, GATA3, a marker of Th2 and innate lymphoid cells type 2 was stained and quantified in the synovial tissue of RA patients. Osteoarthritis patients were used as controls. We found that GATA3^+^ cells were present in the inflamed synovial tissue and were significantly higher in RA than in osteoarthritis patients (Fig. 6a). Moreover, expression of eosinophil peroxidase (EPX), a marker of eosinophils, could be detected in the synovial tissue of RA patients (Fig. 6b). Despite the fact that we did not find any elevation of IL-5 levels in RA patients compared with healthy controls (Fig. 6c), serum levels of EPX were significantly increased in active and inactive RA compared with healthy controls and patients with autoimmunity of RA but no inflammation (‘pre-RA') (Fig. 6d). All together, these data suggest that the cellular components of the Th2–eosinophil response can be found in human inflammatory arthritis as well.

## Discussion

It is well established that the Th2 response is involved in the host defence against helminth infection in mice and humans^20^. In contrast, every littler is know whether and how the Th2 response is involved in immune-inflammatory diseases such as arthritis. This situation is surprising given that genetic and molecular evidence suggests a key role of T cells in several different forms of inflammatory arthritis, such as RA or psoriatic arthritis. Although traditionally being considered as prototype Th1-type disease, more attention has been directed to the role of Th17 cells in inflammatory arthritis in the last years^1^. Nevertheless, therapeutic targeting of Th1- and Th17-derived cytokines has been rather disappointing and only specific forms of arthritis, that is, those linked to psoriasis, appear to respond well to IL-17 pathway neutralization. Hence, the concept that the role of T cells in arthritis may be based on increasing the pro-resolving immune processes is appealing, as Th2-related cytokines such as IL-4, IL-13 and IL-33 have been reported to ameliorate arthritis onset^21^,^22^,^23^,^24^. Furthermore, infection with *Schistosoma* has shown to inhibit inflammatory cytokine production in arthritis^25^. However, whether and how activation of Th2 cells and potentially also eosinophils affect disease still remained elusive.

Herein we provide solid evidence that robust activation of the Th2 responses by *N. brasiliensis* infection counteracts arthritis. Functionally, activation of the STAT6 pathway by IL-4/IL-13 plays a critical role in mitigating and resolving immune activation associated with arthritis. Physiologically, IL-4 and IL-13 have additive effects sharing the same receptor and signalling via STAT6 (refs 19, 26). IL-4/IL-13-mediated activation of the STAT6 pathway is also critical to protect bone and cartilage during arthritis. Osteoclast differentiation, bone loss and cartilage damage were significantly diminished after *N. brasiliensis* infection but not in the respective mutant mice, suggesting that control of arthritis by the IL-4/IL-13/STAT6 pathway is associated with substantial protection from joint damage. These data underline the protective role of Th2-associated cytokines such as IL-4 in inhibition of cartilage damage^27^,^28^ and the supressive effects of IL-4 and IL-13 on murine and human osteoclastogenesis^29^,^30^. In agreement with our observations, constitutive active STAT6 fusion protein was previously shown to decrease osteoclastogenesis and bone destruction^31^.

With respect to the effector pathway, Th2-mediated inhibition and resolution of inflammation appears to depend on the induction of alternative activated macrophages by IL-4 and IL-13 (ref. 32). During arthritis, macrophages preferentially polarize into a pro-inflammatory phenotype. Hence, the number of pro-inflammatory macrophages in the synovium correlates with joint destruction^33^ and is used as a biomarker for clinical response to therapy^34^. Furthermore, pro-inflammatory monocytes are known to be necessary for the induction of K/BxN SIA^18^. In our experiments, we found a robust shift from pro-inflammatory CD11b^+^Ly6C^hi^ monocytes into anti-inflammatory macrophages in the arthritic joints of mice exposed to *N. brasiliensis* infection, suggesting that the key effector cell necessary to maintain arthritis is effectively diminished. Furthermore, the impaired osteoclast differentiation and protection from bone loss can be explained by the alteration of the monocyte status, as osteoclasts preferentially differentiate from pro-inflammatory monocytes^35^, whereas inducers of alternative activated macrophages blunt osteoclast differentiation^24^.

Helminth infection does not only trigger IL-4/IL-13-mediated immune responses important for worm expulsion^36^ but also trigger eosinophil activation. This induction also occurs in the absence of IL-4/IL-13 (ref. 37). In arthritis the increase of eosinophils and IL-5 was associated with resolution of disease, concording with recent study showing the involvement of eosinophils during resolution of inflammation in acute peritonitis or experimental colitis^38^,^39^. Additional experiments using IL-5 triggered eosinophil differentiation or using eosinophil deficient mice provided clear evidence that eosinophils beneficially influence the course of arthritis. These effects could be mediated by production of anti-inflammatory lipids such as PD-1 by eosinophils^40^. These lipids are synthesized by 12/15 lipoxygenase, which has been shown to play a role in the resolution of inflammatory arthritis^41^. Furthermore, IL-33-activated eosinophils could polarize alveolar macrophages towards M2 phenotype in a IL-13-dependent manner^42^.

In summary, our data show that activation of Th2 responses inhibits inflammatory arthritis. Mechanistically, IL-4/IL-13-STAT6 signalling pathway induces macrophage polarization into anti-inflammatory macrophages into the joints. In addition, eosinophils are activated and further contribute to the resolution of disease. These findings are interesting to shape new approaches to rebalance immune homeostasis in arthritis. The presence of Th2 cells and eosinophils in the synovial membrane of RA patients suggests that respective cellular sources to counteract arthritis are also present in human disease. Furthermore, previous data have revealed a Th2 cytokine pattern in very early forms of human RA, when the disease may still be reversible^43^. Hence, activation of Th2 responses and eosinophils in both early and established disease may emerge as a new strategy to treat arthritis.

## Methods

### Mice

Complete *Il4*^−/−^*Il13*^−/−^ mice (4–13ko) (ref. 35), T-cell-specific *Il4*^−/−^*Il13*^−/−^ mice (4–13Tko) (ref. 44), *Stat6*^−/−^ mice^19^, specific eosinophil-deficient ΔdblGATA mice^45^, *Il5*tg mice^46^ and WT mice were on BALB/c background. The hTNFtg mice (strain Tg197 on C57BL/6 background) have been described previously^47^. Clinical assessment of arthritis in hTNFtg mice was started 5 weeks after birth and performed twice a week using a clinical score graded from 0 to 3 (ref. 48). All mice were housed in a temperature- and humidity-controlled facility with free access to food and water. All experiments were performed according to the rules and regulations of the animal facilities FPZ (Franz-Penzoldt-Zentrum, Erlangen) in Germany and approved by the local ethics authorities.

### Helminth infection model

Five hundred third-stage larvae (L3) of *N. brasiliensis* were inoculated subcutaneously at the base of the mice's tail. Mice were provided with antibiotics in the water (2 g l^−1^ neomycin sulfate, 100 mg l^−1^ polymyxin B sulfate; Sigma-Aldrich) for the first 5 days after infection.

### K/BxN SIA model

K/BxN serum transfer arthritis was induced on day 6 after Nb infection by intraperitoneal (i.p.) injection of 200 μl pooled K/BxN serum. The swelling of fore and hind paws were measured daily by digital caliper (Mitutoyo, Japan) and expressed as percentage increase of paw thickness. Development of arthritis was evaluated for each paw using a semi-quantitative scoring system (0–4 per paw; maximum score of 16) as previously described^49^. Mice were killed 7 or 9 days post serum transfer.

### Bone marrow transplantation

To generate bone marrow chimeras, adult *Stat6*^−/−^ mice (CD45.2) were lethally irradiated (11 Gy) and then injected intravenously with 2 × 10^6^ bone marrow cells from WT donor mice (CD45.1). As a control, WT recipient mice (CD45.2) were transferred with 2 × 10^6^ bone marrow cells from WT donor mice (CD45.1) in parallel. After 6 weeks, chimerism was verified by flow cytometry for the appropriate CD45 allele (PE-Cy7-labelled anti-CD45.1 and AlexaFluo700- labelled anti-CD45.2; 1:400; eBioscience).

### IL-5 injection

WT BALB/c mice received daily i.p. injection of 200 ng recombinant murine IL-5 (BD Pharmingen) or equal volumes of vehicle (BSA) alone in 200 μl PBS, starting 1 day before K/BxN serum transfer for 5 days.

### Eosinophil isolation and adoptive transfer

Eosinophils were isolated from the peritoneal cavity of 4get/IL-5tg mice using the Mouse Streptavidin Rapidspheres Isolation Kit and EasySep magnet (Stemcell Technologies), according to the manufacturer's instructions. The following biotinylated antibodies were applied for negative selection: anti CD45R, anti-Ter-119, anti-CD4, anti-CD8, anti-Ly6C and anti-MHC II (all from ebiosience). The purity of eosinophils was determined by flow cytometry as IL-4/eGFP^+^ SiglecF^+^ (PE-labelled anti-SiglecF; 1:400; BD Pharmingen) cells with an average of 90–95%. Isolated eosinophils (2 × 10^7^) or vehicle control was injected intra-orbital into ΔdblGATA mice 5 days after K/BxN serum transfer. Eosinophil transfer was confirmed by flow cytometry gating on IL-4/eGFP^+^ SiglecF^+^ cells in the blood 7 days post serum transfer.

### Histological and morphological analyses

Hind paw and tibia were fixed overnight in 4% formalin and then decalcified in 14% EDTA until bones were pliable. Serial paraffin sections (2 μm) were stained with haematoxylin and eosin and tartrate-resistant acid phosphatase. Area of bone erosion and inflammation, number of osteoclasts per paw, osteoclast surface per bone surface and number of osteoclasts per bone perimeter were assessed by Osteomeasure Analyses System (Osteometrics). The three-dimensional bone structure of tibial bones was measured by a micro-computed tomography scanner (Scanco μCT 35 scanner, Bruettisellen, Switzerland) and analysed by integrated software for segmentation and three-dimensional morphometric analysis.

### Immunohistochemistry

Sections from mouse hind paw and synovial membrane samples from human RA and osteoarthritis patients were deparaffinized, quenched of endogenous peroxidase, blocked with normal serum and incubated with rat anti-mouse major basic protein (provided by Lee's lab in Mayo Clinic), goat anti-human GATA3 (AbD Serotec, UK) and rabbit anti-human EPX antibody (Abcam, UK) at 1:500, 1:3,000 and 1:1,000 dilution, respectively. Slides were washed and incubated with biotinylated secondary IgG and avidin–biotin complex (Vector Laboratories) according to the manufacturer's instructions.

### Quantitative reverse transcriptase–PCR

Total RNA from ankle or knee joint, spleen, mesenteric lymph node and osteoclast culture were extracted by using peqGOLD TRIfast (Peqlab). One microgram of total RNA was reverse transcribed and SYBR Green-based quantitative real-time PCR was performed on Bio-Rad CFX96 Touch Real-Time PCR Detection System. Normalized gene expression values were calculated as the ratio of expression of mRNA of interest to the expression of mRNA for *Actb* (encoding β-actin) or *Hprt* (encoding hypoxanthine guanine phosphoribosyltransferase). The primer sequences are summarized in Supplementary Table 1.

### Flow cytometry

The spleen and mesenteric lymph node were mechanically disrupted and filtered through 40 μm cell strainer, to obtain single-cell suspension. After erythrocytes lysis, 10^6^ cells per well were plated on a 48-well plate and stimulated with leukocyte activation cocktail (BD Pharmingen) for 6 h. Cells were harvested, fixed and permeabilized with fixation/permeabilization buffer (eBioscience) and then intracellularly stained with APC-conjugated IL-4 (1:200; eBioscience), Alexa Fluor488-conjugated IL-13 (1:200; eBioscience) and APC-conjugated IL-5 (1:200; Biolegend). In some experiments, mouse Th1/Th2/Th17 Phenotyping Cocktail (BD Pharmingen, contains PerCP-Cy5.5-labelled anti-CD4, PE-labelled anti-IL-17, fluorescein isothiocyanate-labelled anti-interferon (IFN)-γ and APC-labelled anti-IL-4) was applied according to the manufacturer's instructions. To analyse joints, ankles were cut from 3 mm above the heel until mid-paw, minced and incubated in DMEM medium containing 1 mg ml^−1^ collagenase A (Roche) at 37 °C for 60 min with occasional mixing. Cells were washed, filtered through 40 μm cell strainer, incubated with anti-CD16/CD32 blocking antibody (1: 200; Biolegend) for 10 min at room temperature, followed by staining with antibody cocktail at 4 °C. The following antibodies were used for membrane staining: APC-eFluor780-labeled anti-CD45 (1:400; eBioscience), fluorescein isothiocyanate-labelled anti-CD11b (1:400; BD Pharmingen), PE-labelled anti-SiglecF (1:400; BD Pharmingen), APC-labelled anti-Ly6C (1:800; BD Pharmingen), APC-labelled anti-F4/80 (1:400; Biolegend), PE-labelled anti-PD-L2 (1:400; Biolegend), PerCP-Cy5.5-labelled anti-Ly6G (1:800; Biolegend) and Pacific blue-labelled MHC-II (1:800; Biolegend). Blood was collected into EDTA-containing tubes via cardiac puncture. Erythrocytes were lysed and white blood cells were stained with fluorochrome-conjugated antibodies outlined above. Data were acquired and analysed on FACS calibur (BD Bioscience) and Gallios flow cytometer (Beckman Coulter, Inc.). Live events were collected based on forward and side scatter patterns.

### Serum cytokine levels

Mouse serum level of IL-4, IL-5, IL-10, IL-2, IFN-γ, granulocyte–macrophage colony-stimulating factor and TNF were detected by Mouse Th1/Th2 MULTIPLEX Kit FlowCytomix (eBioscience) according to the manufacturer's instructions. IL-1β and IL-6 level in mouse serum and IL-5 and EPX serum level in patients were analysed by ELISA kit (R&D Systems, Cloud-Clone Corp for EPX), as described in the manufacturer's instructions.

### *In vitro* osteoclastogenesis assay

Total bone marrow cells were isolated from WT BALB/c or 4–13ko mice by flushing femur and tibia. After erythrocytes lysis, cells were incubated overnight with α-MEM supplemented with 5 ng ml^−1^ M-CSF (Peprotech). Non-adherent cells were collected, washed and further cultured in α-MEM supplemented with 10% heat-inactivated FCS, glutamine, 1% penicillin and streptomycin (all from Invitrogen), 30 ng ml^−1^ M-CSF and 10 ng ml^−1^ RANKL (Peprotech) in 48-well plate at the concentration of 1 × 10^6^ cells per ml. In some experiments, 2% serum from Nb-infected or -uninfected mice were added. Medium was changed every 2 days. Osteoclast differentiation was evaluated at day 5 by tartrate-resistant acid phosphatase staining using the leukocyte acid phosphatase kit 386A (Sigma-Aldrich) according to the manufacturer's instructions.

### Patient material

Synovial tissue was derived from knee joints of patients with RA (*n*>10) and OA (*n*>10) (University Hospital of Erlangen-Nuremberg). All samples were fixed in formalin and paraffin embedded. All patients gave written informed consent and their use for research was approved by the Ethics Committees Erlangen. Patient information is listed in Supplementary Table 3.

### Statistical analyses

All data are expressed as mean±s.e.m. The statistical significance was determined by Student's *t*-test for single comparison or analysis of variance test for multiple comparisons using GraphPad Prism software. A *P*-value<0.05 was considered significant.

### Data availability

All relevant data are available from the authors.

## Additional information

**How to cite this article:** Chen, Z. *et al*. Th2 and eosinophil responses suppress inflammatory arthritis. *Nat. Commun.* 7:11596 doi: 10.1038/ncomms11596 (2016).

## Supplementary Material

Supplementary Information Supplementary Figures 1-5 and Supplementary Tables 1-3
