# Supplementary material for: Th2 and eosinophil responses suppress inflammatory arthritis
Source: Nat Commun. 2016 Jun 7;7:11596. doi: 10.1038/ncomms11596 (PMC4899615; doi:10.1038/ncomms11596)
Supplement: Supplementary Information — Supplementary Figures 1-5 and Supplementary Tables 1-3 [file ncomms11596-s1.pdf]

Fig. S1

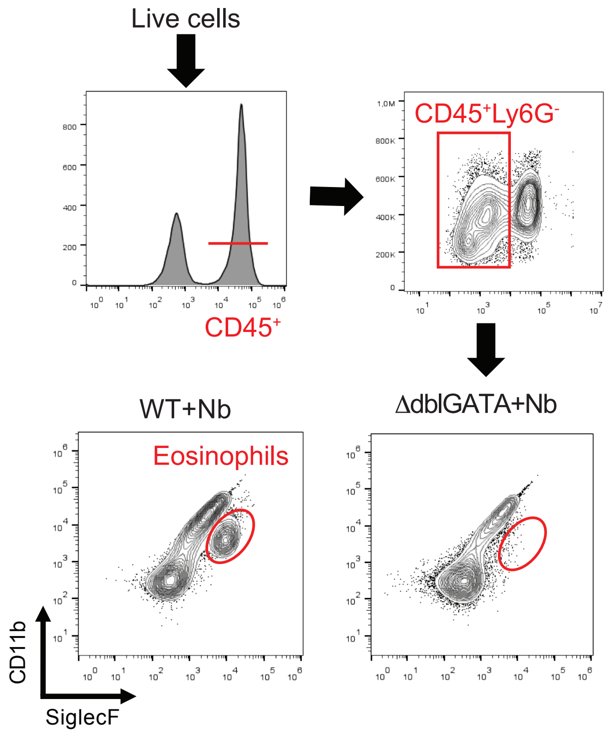

Supplementary Figure 1. Fluorescence activated cell scanner (FACS)- based gating strategy for identification of eosinophils in the joints. Representative contour plots are shown and staining as $CD45^{+}Ly6G^{-}CD11b^{+}SiglecF^{+}$ cells indicated. (WT) wild-type mice; (Nb) challenge with *N. brasiliensis*; (ΔdblGATA) eosinophil deficient mice (control).

Fig. S2

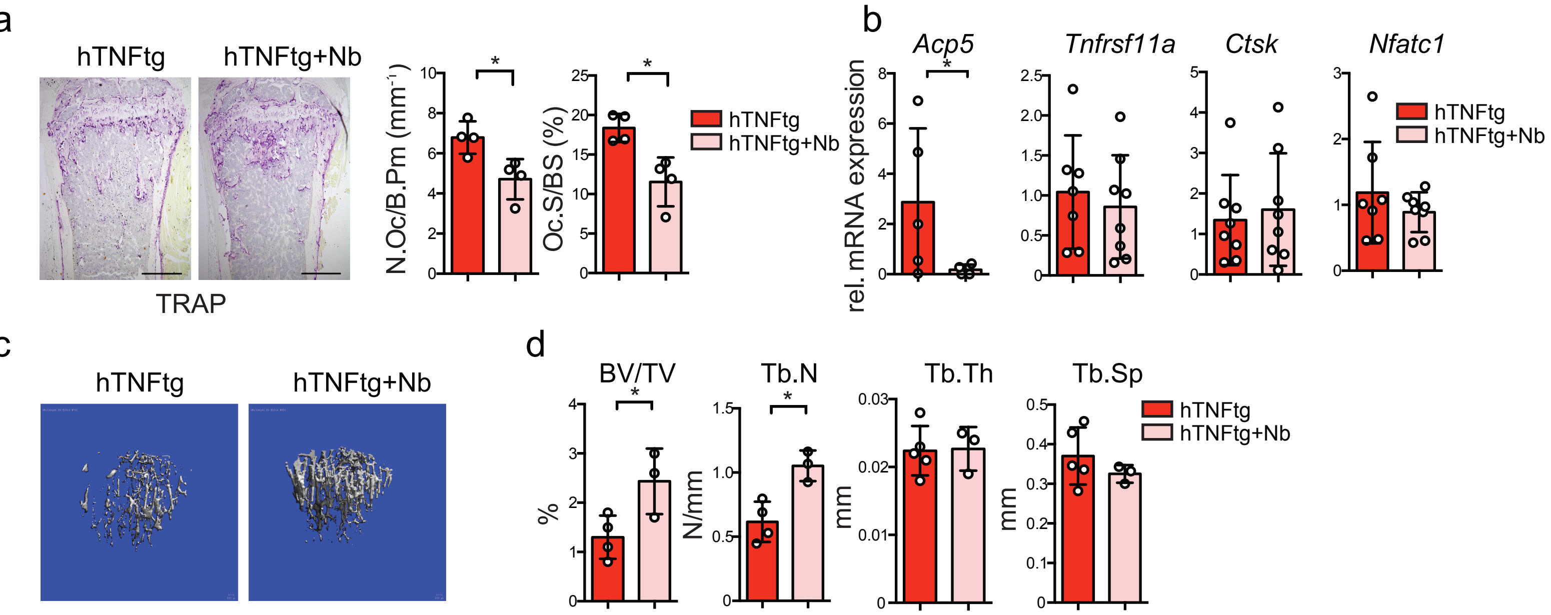

Supplementary Figure 2. (**a**) Tartrate-resistant acid phosphatase (TRAP) staining and quantification of osteoclast surface per bone surface (Oc.S/BS) and number of osteoclasts per bone perimeter (N.Oc/B.Pm) in the tibial bones of human tumor necrosis factor transgenic (hTNFtg) mice with or without *N. brasiliensis* (Nb) challenge (n=4 per group); scale bar: 500µm. (**b**) Quantitative RT-PCR analyses of *Acp5, Ctsk, Tnfrsf11a, Nfatc1* expression in synovial extracts of 9 week-old hTNFtg mice with or without Nb challenge (n=9 per group). (**c**) Representative micro-computed tomography image of the tibial bones and **(d)** quantification of bone microstructure in hTNFtg mice with or without Nb challenge (n=4-5 per group). BV/TV, bone volume per tissue volume; Tb.N, trabecular number; Tb.Th, trabecular thickness; Tb.Sp, trabecular separation. Scale bar: 100µm. Data are shown as mean ± SEM. Asterisks mark statistically significant difference (**P*<0.05 determined by Student's *t* test).

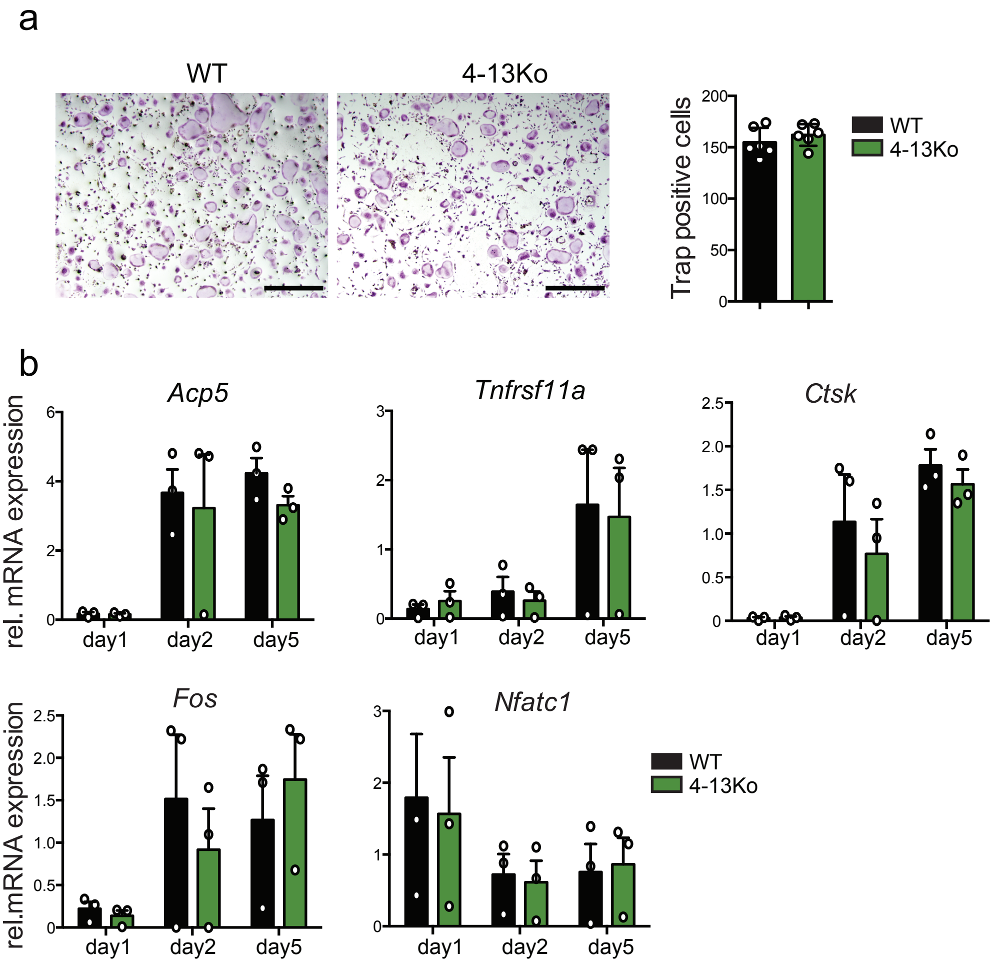

Supplementary Figure 3. (**a**) Quantification of tartrate-resistant acid phosphatase (TRAP) staining and TRAP-positive multi-nucleated osteoclasts from bone marrow monocytes of wild-type (WT) and interleukin-4/interleukin-13 double knockout mice (4-13ko). Data are pooled from two independent experiments and expressed as mean ± SEM (n=6). (**b**) Quantitative RT-PCR analyses of *Acp5, Tnfrsf11a, Ctsk, Fos, Nfatc1* expression in osteoclast culture extract of WT and 4-13ko mice at the indicated time points (n=3 per group).

Fig. S4

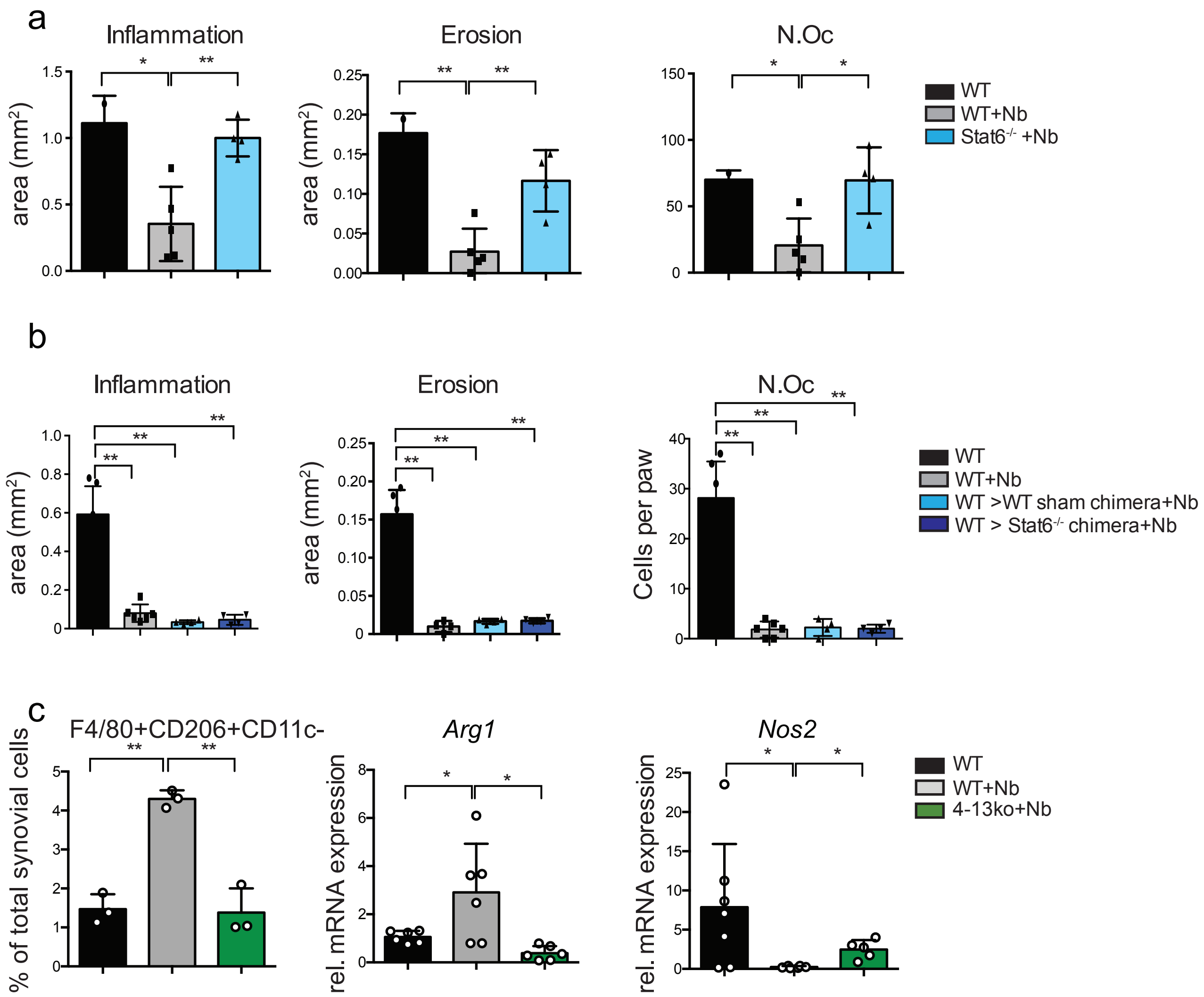

Supplementary Figure 4. (**a**) Quantification of inflammation area, erosion area and number of osteoclasts (N.Oc) per paw in the joints of unchallenged WT mice and Nb-challenged WT and *Stat6*$^{-/-}$ mice 7 days after serum transfer (n=3-5 per group). (**b**) Quantification of inflammation area, erosion area and N.Oc per paw in the joints of unchallenged WT mice and Nb- challenged WT=>WT and WT=>*Stat6*$^{-/-}$ chimeras 9 days after serum transfer (n=5 per group). (**c**) Percentage of anti-inflammatory macrophages (F4/80^{+}CD206^{+}CD11c^{-}) and quantitative RT-PCR analyses of *Arg1* (encoding arginase-1) and *Nos2* (encoding inducible nitric oxide synthase) expression in the joints of unchallenged WT mice and Nb- challenged WT and interleukin-4/interleukin-13 double knockout mice (4-13ko) mice 9 days after serum transfer. Data are shown as mean ± SEM. Asterisks mark statistically significant difference (**P*<0.05; ***P*<0.01; ****P*<0.001 determined by ANOVA test for multiple comparisons).

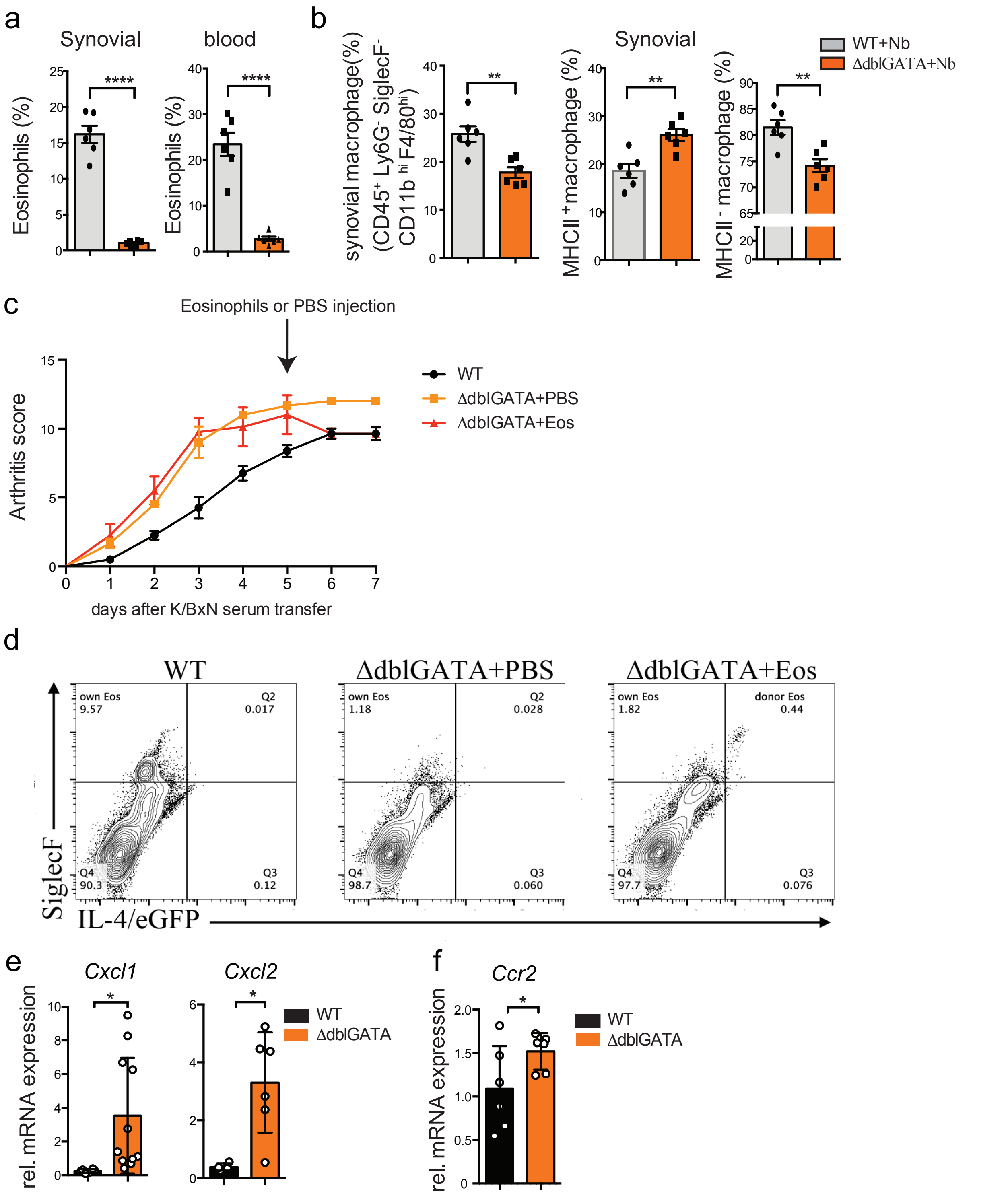

Supplementary Figure 5. (a) Percentage of eosinophils ($CD45^{+}Ly6G^{-}CD11b^{+}$ $SiglecF^{+}$) in the joints and and the blood of Nb- challenged wild-type (WT) and eosinophil-deficient ΔdblGATA mice 9 days after serum transfer (n=6 per group). (**b**) Percentage of macrophages ($CD45^{+}Ly6G^{-}$ $SiglecF^{-}CD11b^{hi}F4/80^{hi}$), MHC II^{+} macrophages and MHC II^{-} macrophages in the joints of Nb-challenged WT and ΔdblGATA mice 9 days after serum transfer (n=6 per group). (**c**) Arthritis scores of WT mice and ΔdblGATA mice injected with vehicle or eosinophils at day 5 after serum transfer (n=6 per group). (**d**) Representative contour plot showing increase of labelled donor eosinophils by flow cytometry gating on IL-4/$eGFP^{+}$ $SiglecF^{+}$ cells in the blood of ΔdblGATA mice 7 days after serum transfer. (**e**) Quantitative RT-PCR analyses of *Cxcl1*, *Cxcl2* expression in the joints of WT and ΔdblGATA mice (n=6-11 per group). (**f**) Quantitative RT-PCR analyses of *Ccr2* expression in the joints of WT and ΔdblGATA mice 9 days after serum transfer (n=6 per group). (**P*<0.05, ***P*<0.01, ****P*<0.001 determined by Student's *t* test)

Supplementary Table 1 The sequences of primers for quantitative RT-PCR

Number	Gene	Forward (5'-3')	Reverse (5'-3')
1	*Actb*	TGT CCA CCT TCC AGC AGA TGT	AGC TCA GTA ACA GTC CGC CTA GA
2	*Hprt*	GTT AAG CAG TAC AGC CCC AAA	AGG GCA TAT CCA ACA ACA AAC TT
3	*Acp5*	GGC CGG CCA CTA CCC CAT CT	CAC CGT AGC CAC AAG CAG GAC TCT
4	*Ctsk*	AGG GCC AAC TCA AGA AGA AAA CT	TGC CAT AGC CCA CCA CCA ACA CT
5	*Tnfrsf11a*	GCC CAG TCT CAT CGT TCT GC	GCA AGC ATC ATT GAC CCA ATT C
6	*Nfatc1*	GAC CCG GAG TTC GAC TTC G	TGA CAG TAG GGG ACA CAT AAC TG
7	*Fos*	CGG GTT TCA ACG CCG ACT AC	CAG GTC TGG GCT GGT GGA GA
8	*Tnf*	CAC AGC CTT CCT CAC AGA GC	GGA GGC AAC AAG GTA GAG AGG
9	*Il1b*	CAG GCA GGC AGT ATC ACT CA	AGG TGC TCA TGT CCT CAT CC
10	*Il6*	TCC TTC CTA CCC CAA TTT CC	GCC ACT CCT TCT GTG ACT CC
11	*Il4*	ATC ATC GGC ATT TTG AAC GAG GTC	ACC TTG GAA GCC CTA CAG ACG A
12	*Il5*	GAT GAG GCT TCC TGT CCC TAC T	TGA CAG GTT TTG GAA TAG CAT TTC C
13	*Nos2*	GAG ACA GGG AAG TCT GAA GCA C	CCA GCA GTA GTT GCT CCT CTT C
14	*Itgax*	CTG GAT AGC CTT TCT TCT GCT G	GCA CAC TGT GTC CGA ACT CA
15	*Arg1*	CTC CAA GCC AAA GTC CTT AGA G	AGG AGC TGT CAT TAG GGA CAT C
16	*Retnla*	CCA TAG AGA GAT TAT CGT GGA	TGG TCG AGT CAA CGA GTA AG
17	*Chil3*	TAC TCA CTT CCA CAG GAG CAG G	CTC CAG TGT AGC CAT CCT TAG G
18	*Mrc1*	CTC TGT TCA GCT ATT GGA CGC	CGG AAT TTC TGG GAT TCA GCT TC
19	*Cxcl1*	TCC AGA GCT TGA AGG TGT TGC C	AAC CAA GGG AGC TTC AGG GTC A
20	*Cxcl2*	CAT CCA GAG CTT GAG TGT GAC G	GGC TTC AGG GTC AAG GCA AAC T
21	*Ccr2*	GCC AGG ACT GAG TGA GAA GG	CAA ATG AGG TTT CCA ACA ACC
22	*Il10*	CGG GAA GAC AAT AAC TGC ACC C	CGG TTA GCA GTA TGT TGT CCA GC

Supplementary Table 2

Absolute numbers Fig. 2 (a-d)

Cell type	WT	WT+Nb	p-value
Eosinophils ($x10^5$)/both ankle	0,245 ± 0,035	0,811 ± 0,099	0,0007
Neutrophils ($x10^5$)/both ankle	0,783 ± 0,039	0,434 ± 0,055	0,0008
synovial Macrophages ($x10^4$)/both ankle	2,315 ± 0,168	2,211 ± 0,259	0,745
$MHCII^+$ Macrophages ($x10^4$)/both ankle	0,860 ± 0,058	0,492 ± 0,069	0,0036
$MHCII^-$ Macrophages ($x10^4$)/both ankle	1,446 ± 0,129	1,716 ± 0,206	0,2974

Supplementary Table 3 Synovial tissue sample list

Patient	Sex	Age	Diagnose
1	female	93	OA
2	female	80	OA
3	female	89	OA
4	female	82	OA
5	female	81	OA
6	female	68	OA
7	female	87	OA
8	female	83	OA
9	male	74	OA
10	female	88	OA
11	female	77	OA
12	female	94	OA
13	female	79	OA
14	male	86	OA
15	female	64	OA
16	female	81	OA
17	female	86	OA
18	female	92	OA
19	female	74	OA
20	female	79	OA
21	male	81	OA
22	female	87	OA
23	female	42	RA
24	female	59	RA
25	female	83	RA
26	female	75	RA
27	female	56	RA
28	female	47	RA
29	male	74	RA
30	female	55	RA
31	female	61	RA
